# Metabolomics-Based Mechanistic Insights into Revealing the Adverse Effects of Pesticides on Plants: An Interactive Review

**DOI:** 10.3390/metabo13020246

**Published:** 2023-02-08

**Authors:** Mohammad Shahid, Udai B. Singh, Mohammad Saghir Khan

**Affiliations:** 1 Plant-Microbe Interaction and Rhizosphere Biology Lab, ICAR-National Bureau of Agriculturally Important Microorganisms (NBAIM), Kushmaur, Mau Nath Bhanjan 275103, Uttar Pradesh, India; 2 Department of Agricultural Microbiology, Faculty of Agriculture Science, Aligarh Muslim University (A.M.U.), Aligarh 202001, Uttar Pradesh, India

**Keywords:** pesticides, toxicological consequences, metabolomic analysis, metabolic pathways

## Abstract

In plant biology, metabolomics is often used to quantitatively assess small molecules, metabolites, and their intermediates in plants. Metabolomics has frequently been applied to detect metabolic alterations in plants exposed to various biotic and abiotic stresses, including pesticides. The widespread use of pesticides and agrochemicals in intensive crop production systems is a serious threat to the functionality and sustainability of agroecosystems. Pesticide accumulation in soil may disrupt soil–plant relationships, thereby posing a pollution risk to agricultural output. Application of metabolomic techniques in the assessment of the biological consequences of pesticides at the molecular level has emerged as a crucial technique in exposome investigations. State-of-the-art metabolomic approaches such as GC–MS, LC–MS/MS UHPLC, UPLC–IMS–QToF, GC/EI/MS, MALDI-TOF MS, and ^1^H-HR-MAS NMR, etc., investigating the harmful effects of agricultural pesticides have been reviewed. This updated review seeks to outline the key uses of metabolomics related to the evaluation of the toxicological impacts of pesticides on agronomically important crops in exposome assays as well as bench-scale studies. Overall, this review describes the potential uses of metabolomics as a method for evaluating the safety of agricultural chemicals for regulatory applications. Additionally, the most recent developments in metabolomic tools applied to pesticide toxicology and also the difficulties in utilizing this approach are discussed.

## 1. Introduction

The current review gives a general overview of how metabolomics is now used to evaluate the phytotoxic effects of pesticides and their biological responses. Even though metabolomics can be used to study diverse biologicals, this review focuses on research involving plants because they are highly sensitive to many abiotic stresses (such as heavy metals, salinity, drought, water logging, temperature, oxidative stress, and other organic contaminants) including chemical pesticides. This review specifically aims to highlight the use of various analytical techniques and reveal metabolite changes in plants while growing in a stressed environment.

## 2. Pesticide Toxicity to Agricultural Crops: A Burning Problem

According to the US EPA (United States Environmental Protection Agency; William Jefferson Clinton Federal Building, Washington, DC, USA), “any substance or mixture of substances intended for preventing, destroying, repelling, or mitigating any pest” falls under the category of pesticides [1]. Pesticides comprise herbicides, fungicides, insecticides, rodenticides, molluscicides, nematicides, and plant growth regulators. Among pesticides, herbicides have long been employed to control unwanted weeds that inhibit plant development [2,3]; insecticides control/destroy a variety of insect pests [4]; and fungicides are used to prevent fungal pathogens from damaging agricultural crops [5,6]. There are currently several environmental media, including food, water, and soil, that contain pesticide residues [7]. Uncontrolled use of pesticides/agricultural chemical protectants often causes toxicity to plants, leading to the retardation in growth, physiology [8], biochemical processes [9], yield attributes [10], and nitrogen and carbon metabolism [11]. Physiologically, pesticides are reported to hinder the photosynthesis process by adversely impacting plant photosystems [12]. In a study, the pesticide hexaconazole has been shown to shorten the length of plant organs (roots and shoots), fresh weights, protein content, and chlorophyll pigment in *Vigna radiata* (L.) raised in soil treated with increasing concentrations of the pesticide [13]. Additionally, pesticides are reported to cause oxidative stress in plants by producing reactive oxygen species (ROS) such as superoxide anions (O^−2^) and hydrogen peroxide (H_2_O_2_) [14,15]. For instance, the pesticide chlorothalonil enhanced H_2_O_2_ levels in tomato plants grown under pesticide stress [15]. The ROS that are subsequently deposited inside plant tissues impair the integrity of the membrane, ultimately causing electrolytes to flow outside the cell [16]. Plants eventually die due to a loss/imbalance of vital ions from the interior to the exterior environment. However, plants, including tomato, while growing under pesticide stress have RBOH1 (respiratory burst oxidase homologous 1) that eventually controls the levels of H_2_O_2_ and allows plants to grow normally [17]. Additionally, chemical pesticides alter root physiology and the morpho-anatomical features of plants [18]. For instance, higher concentrations of neonicotinoid insecticides (imidacloprid and thiamethoxam) have been shown to decrease the biological growth features, symbiotic attributes, and nutrient uptake in chickpeas [*Cicer arietinum* (L.)] [8]. A microscopic examination of insecticide-stressed roots revealed obvious damage/alteration in surface morphology/root tips and anatomical structure [8]. In a related work, pyrimorph (a novel fungicide which inhibits the majority of fungal pathogens) seriously impaired plant physiological machinery by substantially inhibiting the chloroplasts’ electron-transport (ET) reactions [19]. Apart from these, chemical pesticides have a variety of other harmful effects on plant cells, such as chelation and the development of mixed disulfide bonds that are transported across membranes [20,21]. They also operate as multiple-site inhibitors. Furthermore, according to reports, pesticides increase the ratios of NAD and NADP (NAD/NADP), which interferes with the electron transport system (ETS) and raises ATP levels by causing changes in the enzyme system, preserving chlorophyll and leaf and grain protein in plants [22]. Additionally, pesticides alter the effective quantum yield (PS-II) and maximal quantum efficiency (Fv/Fm) of PS-II of plants [23].

Similar to the toxic impact of agrichemicals on plants, the hazardous effects of pesticides on non-target organisms have also drawn considerable attention worldwide because pesticide application in cropping systems is increasing alarmingly and consistently [24]. Although several pesticides were outlawed because of their ecotoxicity and environmental persistence, pesticides that are still present in the environment may nevertheless have an impact on agronomically useful, non-target organisms. Due to these and other reasons that are not covered in this review, analytically based metabolomics studies have become pertinent in understanding the actual pesticide-induced metabolic changes in agronomically important crops.

## 3. Pesticidal Toxicity Mechanisms: An Overview

According to the existing literature dealing with the hazardous behaviour of pesticides starting with absorption by leaves and roots and progressing to translocation and internalization in various plant organs, the phytotoxic events of pesticides leading to plant death can be categorized as follows: (i) adsorption: chemical/agricultural pesticides adhere to the plant surfaces, leaves, and roots due to different forces (attractive and repulsive); (ii) uptake: following foliar/soil application, pesticides are intracellularly accumulated/taken up by different plant organs; (iii) internalization: following effective adsorption, pesticides are internalised (infiltrated) and subsequently deposited onto various cellular organelles (vacuoles and tonoplast); and (iv) translocation: pesticides move from one plant organ to another via circulatory tissues, such as the xylem. Intracellular connections enable the transport of these harmful substances from cell to cell throughout the plant system. With their sequestration on the nuclear membrane, pesticides begin to disturb cellular homeostasis by (a) deteriorating the nuclear components; (b) dissipating the mitochondrial membrane potential (ΔΨm); (c) disrupting homeostasis; (d) causing genotoxicity (as evidenced by the disruption of mitosis (mitotic index) and development of chromosomal abnormalities, pesticides cause genotoxic effects and damage the DNA); apoptosis is also indicated by the fact that pesticides cause the nuclear DNA (sub-G1 phase) to degrade in a caspase-dependent manner; (e) generating reactive oxygen species (ROS) (pesticides upsurge the lipid peroxidation and production of ROS (O_2_^−^, OH, and H_2_O_2_), which is the ultimate cause of mitochondrial membrane potential); (e) causing a loss of physiological and metabolic processes resulting in a decrease in biological characteristics and crop production; and (f) affecting the mortality of plants due to one or more simultaneous activities of pesticides. Despite these reports, more detailed research focussing on crop-specific pesticides is needed to understand how pesticides cause phytotoxicity. In this context, some state-of-the-art molecular techniques, such as metabolomics, proteomics, and genomics, are anticipated to improve our understanding about the phytotoxicity of agricultural pesticides.

## 4. Metabolomics: A Brief Outline

**Figure 1:**
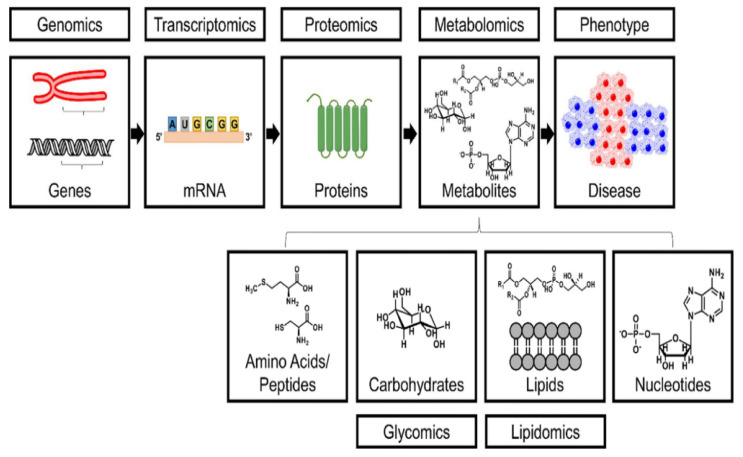
A simplified overview of several “omics” methodologies. Analysing metabolites, including amino acids, peptides, carbohydrates, lipids, and nucleotides that vary in response to biological disturbances which may show up as various environmental changes, is the focus of the field of metabolomics. Adapted with permission from Matich et al. [30].

The field of science known as “metabolomics” is devoted to the assessment/measure (both qualitative and quantitative) of the metabolites found in biological materials such as cells, tissues, and other plant organs [25]. The metabolome, also referred to as the entire collection of metabolites present in a given biological sample, is influenced by endogenous variables and the cellular response to environmental cues [26]. Among various metabolomic approaches (Figure 1), environmental metabolomics reveals how living things interact with their surrounding environment [27]. Compared to the transcriptome and proteome, the metabolome is significantly more closely related to the cellular phenotype [28]. In response to gene expression and protein activity, metabolites, substrates, cofactors, and products of enzymatic reactions are secreted, transported, degraded, and stored. Additionally, changes in metabolite levels can have an impact on other “omics” layers, such as proteomics and transcriptomics. As an illustration, lipids are an energy source and can function as second messengers in cellular signalling cascades, whereas amino acids and nucleotides are the chemical building blocks of nucleic acids and proteins [29]. As a result, since metabolite levels reflect the cellular state, characterization of the metabolome becomes essential for comprehending the phenotype of the target species and the physiological mechanisms that are activated under particular environmental conditions.

Currently, metabolomics is employed extensively in the field of pesticide toxicology. In laboratory-level research, the inadvertent effects of several pesticides on plants’ metabolomes were identified using various metabolomic approaches. For instance, lindane and chlordecone, two organochlorine pesticides, were examined for their effects on *Zea mays* (L.) roots using ^1^H-NMR-HRMAS-based metabolomics. During analysis, approx. 26 altered metabolites were identified, which were then mapped to eight metabolic pathways in the Plant Metabolic Network and Kyoto Encyclopaedia of Genes and Genomes [31,32]. The findings revealed that sucrose was downregulated, pointing to variations in the metabolism of starch and sugar. Succinate and fumarate are the two tricarboxylic acid (TCA)-cycle metabolites that underwent alteration, suggesting that the roots may be respiring less quickly. Additionally, considerable changes in the amino acids gamma-aminobutyric acid, asparagine, and isoleucine were noticed. These findings point to an imbalance between carbon (C) and nitrogen (N) under exposure to organochlorine pesticides, as well as an increase in oxidized fatty acids, which points to a degraded state of cell wall integrity and vitality [30]. Similarly, in a comparable study, ascorbate, sugar, lipid, nucleotide, and amino acid metabolism was altered when *Lactuca sativa* plants were exposed to the fungicide mancozeb [33]. Rice (*Oryza sativa* L.) is another agronomically important cereal food crop that has undergone extensive metabolomics research. Using GC–LRMS analysis, metabolite alterations in rice plants following the exposure of the insecticide diazinon were recorded. Treatment had time-dependent effects, including changes in metabolites involved in the biosynthesis and metabolism of sugars, amino acids, and organic acids, alterations in antioxidant defence, disruption of energy metabolism, and oxidative stress [34]. Zhao et al. [35] used a GC–LRMS-targeted metabolomics technique to investigate how pesticides, transgenes, and backcross breeding affected rice leaves and seeds. The findings revealed that seeds and leaves of *O. sativa* (L.) had up-regulated levels of amino acids, while rice seeds had downregulated levels of phenols, antioxidants, and carbohydrates.

## 5. Analysis Processes of Metabolomics

Metabolomics, a crucial component of systems biology, helps elucidate the connections between various metabolites and the associated physiological and pathological states of plants by quantitatively analysing changes in small-molecule metabolites in plant tissues [36]. Currently, nuclear magnetic resonance (NMR) and mass spectrometry (MS) are the two primary analytical methods utilized in environmental metabolomics [37]. The use of NMR as an analytical tool in metabolomics dates back to the early 2000s [38]. It is a quick and incredibly repeatable spectroscopic technique that relies on the atomic nuclei’s ability to absorb energy and release it again in response to changes in external magnetic fields [39]. NMR can gather comprehensive information about the target samples by detecting most endogenous metabolites in plant samples [40]. The benefits of NMR include the results of its detection being consistently stable and repeatable [41]. Additionally, the NMR metabolome-based sample pretreatment technique is rather straightforward, and the sample may be evaluated and identified objectively and non-destructively [42]. The most popular platform for analysing metabolomics data is the mass spectrometer (MS), which may gather spectral information about the relative strength of the measured chemical’s mass-to-charge ratio (*m*/*z*) [43]. As of now, GC–MS and LC–MS are the two most widely utilized MS-based methods in environmental metabolomics. LC–MS can evaluate molecules that are highly polar, have a high relative molecular mass, are thermally unstable, or are not easily volatile [44]. Additionally, derivatization is not necessary for sample preparation for LC–MS-based metabolomic analysis [45]. However, its drawbacks are the prolonged processing time and the absence of appropriate databases. Contrarily, the primary benefit of GC–MS is that it has an established database, which facilitates the easier characterization of metabolites [46]. However, this approach is more sensitive, particularly for metabolites containing volatile compounds.

### Preparation of Samples for Metabolomic Evaluation

In order to keep up with the expansion in biological studies, metabolomics has attracted attention as a quicker and more thorough analytic technique. When extracting metabolites, pre-treatment of biological samples is necessary because the abundance of some metabolites or contaminants might make it difficult to identify other metabolites [47]. The development of a successful metabolite extraction procedure is, therefore, essential in every metabolic profiling study. Since internal or external environmental changes cause low-molecular-weight metabolites to degrade, quick quenching of metabolic enzymes is one of the most crucial requirements for precise analysis [48]. For instance, rapid freezing in liquid nitrogen is frequently employed to fully block metabolic events [49]. In practice, the quenching and metabolite extraction processes are carried out concurrently. An effective method for quenching and extracting metabolites is to use an organic solvent such as methanol, chloroform, isopropanol, acetonitrile, acetone, ethanol, or hexane, etc. [50]. Methanol and aqueous alcohol are popularly used solvents for identifying polar metabolites [51]. However, plants are adversely affected by the constant use of solvents, which causes the majority of their organs to perish. Despite the fact that organic solvents such as acetone, DMSO, and DMF, as well as aromatic chemicals such as benzene, toluene, and chlorinated solvents, cause environmental pollution, they are nevertheless utilized extensively [52]. The primary goal of green chemistry is to utilize fewer solvents or to replace them with less harmful ones. The category of “green solvents” includes water, supercritical fluids, and non-toxic liquid polymers such as PEG [53]. They are categorized according to their ease of accessibility, low toxicity, and potential for reuse. Because of their diverse behaviour, including excellent stability, biodegradability, low vapor pressure, and low toxicity, ionic liquids (ILs) are the most well-liked among scientists [54]. Because of these characteristics, ILs are the ideal alternative to conventional chemicals and are seen as green solvents [55]. Additionally, pressured liquid extraction (PLE), often referred to as accelerated solvent extraction (ASE), pressurized solvent extraction (PSE), or pressurized fluid extraction (PFE), is a technique for extracting solid and semi-solid materials using liquid solvents in metabolomics [56]. Furthermore, the method of extracting one component (the extractant) from another (the matrix) using a supercritical fluid as the extracting solvent is known as supercritical fluid extraction (SFE) [57]. Though it can also be accomplished with liquids, extraction is typically achieved from a solid matrix. SFE can be employed as a step in the preparation of samples for metabolomic analysis. Eutectic solvents are a combination of a hydrogen bond acceptor (HBA) and a hydrogen bond donor (HBD) at room temperature [58]. The interaction between HBDs and other molecules, such as sugars, amino acids, carboxylic acids (such as benzoic acid, citric acid, or succinic acid), or amines (such as urea or benzamide), is crucial for the synthesis of these solvents.

For lipid metabolites, several conventional techniques including the Folch method and Bligh and Dyer methods are frequently employed [59]. Polar metabolites may be extracted from lipids and pigments using similar extraction techniques, and they can then be separated from one another [60]. Intracellular metabolites from plant samples are isolated after cell destruction using enzymatic (such as lysozyme) or physical methods, bead beaters, ultrasonication, and freeze–thaw cycles [61]. Following extraction, the extracts are concentrated. Due to high volatility and potential loss during the concentration stage, some metabolites, such as short-chain organic acids, should be handled carefully [62].

For environmental metabolome analysis, preparation of samples is primarily influenced by analytical instrumentation, followed by the chemical characteristics of the sample [63]. In this regard, various workers have used different solvents, methods of metabolite extraction, and metabolomic tools to investigate the effect of environmental variables on plant samples (Table 1). For instance, Maia and co-workers [64] developed a more effective metabolite extraction method for the analysis of the metabolic profile of grapevine leaves by means of DI–FTICR–MS-based metabolomics. According to their method, both polar and non-polar molecules containing all significant classes present in plants and enhancing the metabolome coverage can be extracted.

**Figure 2:**
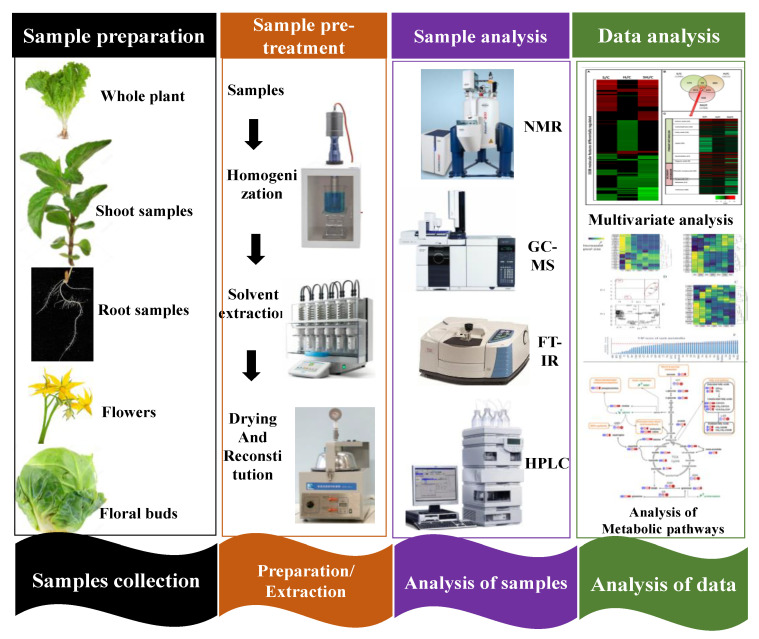
The overall scheme of biological sample analysis using metabolomic tools.

To determine the optimal method for preparing samples for NMR analysis, Brown et al. [65] investigated several solvents, including times for depuration and lyophilization. For the extraction, they used a number of solvents such as a D_2_O-based phosphate buffer, CH_3_CN, dimethyl sulfoxide (DMSO), chloroform, methanol, etc. They suggested that phosphate buffers (based on D_2_O) have the best repeatability, the highest concentration, and the widest range of metabolites, offering the most comprehensive metabolic profile. Additionally, lengthier depuration times resulted in less fluctuation in the results, and they discovered minimal changes between the data of homogenized tissue samples before lyophilization and those lyophilized individually [66]. Additionally, McKelvie et al. [67] analysed various sample derivatization methods such as HMDS, TFA, hydroxylamine hydrochloride, MSTFA, and MTBSTFA for GC/MS analysis. Figure 2 depicts the overall scheme of biological sample analysis using metabolomic tools.

**Table 1:** Examples of some extraction methods for metabolomic analysis of plants.

Plant	Organs Used/Involved	Extraction Solvents	Methods of Extraction	Metabolomic Tools/Platform	References
*Chenopodium album* (L.)	Leaves, stem	Methanol	Lyophilization + centrifugation	UHPLC-QQQ-MS	[68]
*Lemna minor* (L.)	Leaves	Methanol-ethyl acetate mixture (50:50, *v*/*v*)/Ribitol	Extraction, pulverization, sonication	GC/EI/MS	[69]
*Zea mays* (L.)	Root	CH_3_OH:CHCl_3_ (2:2, *v*/*v*)	-	^1^H-HRMAS NMR analysis	[70]
*Lonicerae japonicae* flos	Flower buds	Methanol	Ultrasonication	UPLC/Q-Orbitrap-Full MS	[71]
*Lactuca sativa* (L.)	Leaf tissues	Methanol (80%) and formic acid (0.1%)	Homogenization	UHPLC	[72]
*Arabidopsis thaliana* (L.)	Plant cells	Methanol and H_2_O	Homogenization	LC/EI/MS	[73]
*A. thaliana* (L.)	Leaves	Methanol, chloroform, and H_2_O (chilled)	Homogenization	LDI/MS	[74]
*Cucumis sativus* (L.)	Leaves	Methanol/water (100:0), acetonitrile/water (80:20) acetone/water	Homogenization+ centrifugation	LS–MS/MS	[75]
*Solanum lycopersicum* (L.)	Leaves	Methanol (70%)	Centrifugation	UHPLC/Q-TOF	[76]
*Solanum tuberosum* (L.)	Tubers	Methanol	Homogenization	UPLC-IMS-QtoF	[77]
*Oryza sativa* (L.)	Leaves and seeds	Acetonitrile/isopropanol/water (3/3/2, *v*/*v*/*v*)	Centrifugation	GC–MS	[78]
*O. sativa* (L.)	Leaves	Methanol	Solvent extraction + homogenization	GC–MS	[79]
*S. lycopersicum* (L.)	Fruit	Acetonitrile/acetic acid/anhydrous MgSO_4_/sodium acetate	Solvent extraction	LC–MS	[80]
*O. sativa* (L.)	Leaves	Methanol and H_2_O	Solvent extraction + derivatization/homogenization	HS-SPME/GC-MS	[81]
*C. sativus* (L.)	Fruit	Methanol/ H_2_O and chloroform	Extraction/homogenization	UHPLC-Q-Orbitrap-HRMS	[82]
*O. sativa* (L.)	Leaves	Methanol/methyl-tertiary butyl ether/H_2_O	Solvent extraction/homogenization	LC–MS	[83]
*Tasmannia piperita* (L.)	Leaves	Methanol	Solvent extraction	UHPLC-HRMS	[84]
*Vitis vinifera* (L.)	Leaves	Methanol/chloroform/H_2_O	Solvent extraction/fractionation/homogenization	FTICR–MS	[64]
*O. sativa* (L.)	Leaves	Acetonitrile/ isopropanol/water (3:3:2, *v*/*v*/*v*)	Solvent extraction + ultrasonication	GC–MS	[85]
*Glycine max* merr	Leaves	Methanol/acetonitrile/deionized water, 2/2/1, *v*/*v*/*v*	Ultrasonication/ centrifugation	LC–MS/MS	[86]
*Helianthus annuus*	Plants	Perchloric acid	-	1D ^1^H NMR	[87]
*Brassica oleracea*	Leaves	Methanol/chloroform/water in a 2:2:1	Solvent extraction+ centrifugation	NMR	[88]
*L. sativa* (L.)	Leaves	Acetone: hexane (1:1, *v*/*v*)	Solvent extraction	GC×GC–MS	[89]
*Beta* *vulgaris* (L.)	Roots	Ethanol	Solvent extraction/ homogenization	UPLC Q-TOF LC-MS	[90]

## 6. Metabolomics Approaches Used to Assess Pesticide–Plant Interactions

Environmental metabolomics offers its relevance and potentiality in investigating the effect of pollutants/contaminants on biological systems, including plants, and in the evaluation of environmental health and safety. The range of metabolites covered by metabolomics has expanded due to developments in analytical instrumentation. The two most used analytical methods are mass spectrometry (MS) combined with chromatographic separation and nuclear magnetic resonance (NMR) spectroscopy. A detailed description of some of the metabolomic tools used in assessing the phytotoxic impact of pesticides on the plant metabolome has been discussed in the following section (Table 2).

### 6.1. Nuclear Magnetic Resonance (NMR)-Based Metabolomics

The nuclear magnetic resonance (NMR) technique was firstly utilised in 1974 to analyse the metabolome [91]. This technique provides sample handling convenience and quantitative power, allowing for the avoidance of metabolite extraction processes [92]. Additionally, NMR is a highly reliable and non-destructive metabolomic tool [93]. NMR can detect the majority of endogenous metabolites in an organism and thus provide comprehensive information about the target biologicals [94]. This method, however, harbours many difficulties related to automation and high-throughput analysis and requires large concentrations, usually in the millimolar (mM) range [95]. The varied nuclei (for example, ^1^H, ^13^C, ^5^N, and ^31^P) and the degree of correlation that they offer can change the type and variety of NMR investigation. To help in metabolite identification, two-dimensional (2D) approaches seek to increase sensitivity, shorten acquisition times, and give more structural detail [96]. Additionally, the high-resolution magic-angle spinning (HR-MAS) NMR is capable of analysing both liquid samples and intact tissue samples. Several NMR studies have been used to evaluate the response of pesticides to crop plants. In this regard, root tips of *Zea mays* (L.) exposed to 2.5 to 25 µM of lindane and chlordecone were assessed using ^1^H-HR-MAS NMR. The results revealed that pesticide exposure increased the amount of fatty acids (FA) present in root tissues, which was accompanied by a significant rise in oxidised FAs. Furthermore, pesticidal stress caused an increase in LOX3 transcription levels due to a build-up of asparagine and oxidised fatty acids, which induced protein and lipid catabolism [70]. In another HR-MAS NMR-based study, Pereira et al. [33] demonstrated that mancozeb negatively affected the metabolomic profile of *Lactuca sativa* (L.). Mancozeb induced oxidative stress in crops by causing a variation in phenylalanine (PPA) and polyphenols (PPO). Furthermore, pesticide exposure led to alterations in amino acids leading to up-regulation in the Krebs cycle. Abnormalities in sucrose, phospholipid, nucleotide, and nicotinamide metabolism were also observed.

### 6.2. MS-Based Metabolomics

#### 6.2.1. Gas Chromatography–Mass Spectrophotometry (GC–MS)-Based Metabolomics

**Figure 3:**
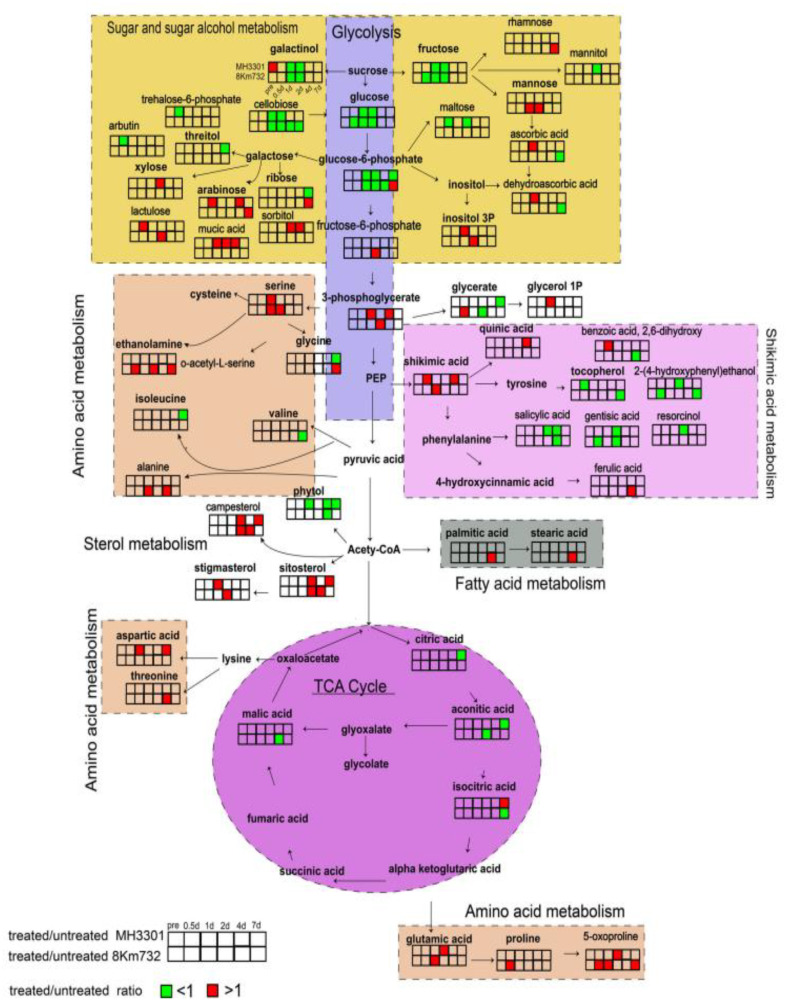
Pathway analysis of differential metabolites related to pesticide stress of rice leaves (MH3301 and 8Km732). The metabolic response indicates relative metabolite levels of the treated group against the control group (treated/untreated) at the same time point. Red (treated/untreated > 1) and green (treated/untreated < 1) represent a *p*-value of metabolite less than 0.05 between treated and untreated groups, while white reflects a *p*-value more than 0.05 (colour figure online). Adapted from Zhao et al. [35] Springer Nature Publishing group 2022.

Due to its excellent sensitivity, detection limits of very small molecules, versatility for high-throughput analysis, and a variety of application capabilities, MS is considered one of the most frequently employed analytical technologies in the area of environmental metabolomics [97,98]. When using standard or other reference substances, such as surrogates, MS-based metabolomics, which is based on tracking the mass-to-charge ratios (*m*/*z*) of all ionizable molecules present in the sample, can provide absolute quantification as well as relative metabolite levels between samples [99,100]. Due to the enormous chemical diversity among metabolite classes, different separation techniques, such as liquid chromatography (LC), gas chromatography (GC), and capillary electrophoresis (CE), have been connected to the mass spectrometer (MS) [101]. These separation methods depend on variations in the boiling temperatures of volatile and non-volatile chemicals, as well as the ionic mobility of charged ions. LC–MS and GC–MS are the most frequently used MS-based methods due to their ability to cover a large portion of the metabolome. However, features such as superior chromatographic resolution, repeatable retention durations, and simplicity of use make GC–MS a more preferable technique over LC–MS. In addition, GC–MS requires less maintenance than other methods and can deliver higher throughput [102]. Furthermore, GC is frequently connected to hard ionisation sources such as electron impact ionisation (EI), enabling in-source fragmentation and identification of molecular ions with the vast databases available for GC–MS [103]. GC–MS can only be used to examine volatile analytes; it cannot be used to detect non-volatile substances such as amino acids, sugars, and organic acids without first derivatizing them [104]. Several MS-based metabolomic studies have been performed to assess the phytotoxic impact of agricultural pesticides on different crops. For instance, very recently LC–MS/MS analysis of acetamiprid (ACE)- and cyromazine (CYR)-exposed *Vigna unguiculata* (L.) plants revealed the modifications/alterations in the metabolome of leaf tissues. Both the pesticides resulted in a significant alteration in the metabolism of amino acids. Furthermore, the flavonoids and sugars synthesis pathways were changed by CYR. Additionally, phenylalanine, isoleucine, and glutamate levels, as well as expressions of genes related to these amino acids, were significantly lowered. Plants exposed to ACE had higher anthocyanin levels and lower levels of quercetin and naringenin chalcone [105]. Likewise, Cu-based pesticides caused the depletion of antioxidants in *Cucumis sativus* (L.), which increased the levels of benzoic acid, gallic acid hydrate, and p-coumaric acid, suggesting the activation of a defence mechanism [75]. Similarly, Liu and Zhu [78] performed GC–MS analysis in order to evaluate the metabolic profiling of chlorpyrifos-exposed *Oryza sativa* (L.) leaves. They found that pesticide application dramatically altered the amino acid and key DEG (mainly enriched in aspartate and glutamate metabolism) metabolic pathways. In addition, chlorpyrifos caused the degradation of soluble proteins (48.7% reduction over the control). In addition, Zhao et al. [35] used a GC–LRMS-targeted metabolomics technique to investigate how pesticides, transgenes, and backcross breeding affected rice leaves and seeds. The findings revealed that seeds and leaves of *O. sativa* (L.) had up-regulated levels of amino acids, while rice seeds had downregulated levels of phenols, antioxidants, and carbohydrates (Figure 3).

#### 6.2.2. Liquid Chromatography–Mass Spectrophotometry (LC–MS)-Based Metabolomics

LC–MS is one of the most popular and widely used analytical techniques that, when applied in metabolomics, provides spectral information about the relative intensity of the mass to-charge ratio (*m*/*z*) of the measured chemicals. Non-volatile substances can also be analysed using LC–MS, which offers great sensitivity and selectivity. However, when the sample matrix is complicated, and depending on the type of LC column used, separation via LC is sensitive to retention time fluctuations [106]. To reduce interference from complicated matrices, the use of 2D LC and GC has also been applied to metabolomics. Chromatographic resolution and peak capacity have both been improved because of this two-dimensional method [107,108]. Electrospray ionisation (ESI) and a high-resolution mass spectrometer (HRMS), which enable highly accurate mass measurements of the precursor and fragment ions for metabolite identification, are frequently used in LC–MS analyses [109,110]. The smallest variation in *m*/*z* that can be distinguished for a particular signal (at a given *m*/*z* value) is referred to as resolution ^®^ in MS terminology. In the area of plant biology, this technique is often used to assess the influence of toxic pollutants, including agricultural pesticides, on metabolomic profiling of various crops. For instance, in a study, Danek et al. [111] applied different pesticides such as deltamethrin (DMN), thiamethoxam (THIA), metalaxyl (MTL), and cyhalothrin (CLN) and their metabolites to *Raphanus sativus* var. *longipinnatus*, which was exposed to these compounds under experimental conditions employing LC–MS/MS. After harvesting, analysis revealed that *R. sativus* contained MTL (0.008 mg/kg), metalaxyl acid (0.009 mg/kg), and (+)-trans-chrysanthemic acid (0.098 mg/kg). Even though pesticide concentrations were below the analytical method’s limit of detection (0.005–0.006 mg/kg), non-targeted analysis showed the existence of THIA, CLN, and DMN metabolites in pesticide-treated plants. In addition, tyramine and leucine (non-specific) and serotonin, tryptamine, dopamine, and epinephrine (specific) metabolites were detected. The LC–MS/MS technique, therefore, demonstrated the importance of non-targeted analysis as a method for assessing pesticide exposure in plants, even after the parent molecule has been entirely metabolized. LS–MS analysis was carried out to compare the glyphosate and triclopyr susceptibilities of several *Conyza* spp. (a flowering plant) biotypes [112]. Significant variations in glyphosate content in plant samples were noticed. This resistant biotype has a faster and more powerful metabolism than sensitive biotypes, converting glyphosate into the metabolites amino methylphosphonic acid (AMPA), glyoxylate, and sarcosine, thereby lowering the amount of intracellular glyphosate that reaches the target enzyme EPSPS [113]. The residues and dissipation of imidacloprid in *Ipomoea batata* (L.) and *Zizania latifolia* (L.) were investigated using liquid chromatography–tandem mass spectrometry (LC–MS/MS). First-order kinetics were used to describe imidacloprid dissipation dynamics in plants, with half-lives ranging from 3.2 to 5.5 days in each sampling location. In Z. latifolia and purple sweet potatoes, the terminal imidacloprid residues ranged from 0.005 to 0.120 mg kg^−1^, and average insecticide recovery in both crops ranged from 82.12 to 113.79%. Likewise, in order to identify 60 pesticides and their residues, 144 vegetable and fruit samples were analysed using the LC–MS/MS method [114].

#### 6.2.3. Gas Chromatography/Electron Impact Mass Spectrometry (GC/EI/MS)-Based Metabolomics

In environmental studies, the GC/EI/MS metabolomics technique has been used to research a variety of subjects, including monitoring the hazardous effects of pesticides on diverse crop plants [115]. Herbicides, glyphosate, and metribuzin are frequently employed in agricultural practises, and residues from both have been found in water samples at amounts ranging from ppb to ppm. In this context, Kostopoulou et al. [69] used the aquatic plant *Lemna minor* L. (duckweed) to assess the GC/EI/MS metabolomics analysis for investigation of the combined impacts of glyphosate and metribuzin and the evaluation of the health and safety of aquatic ecosystems. Duckweed is often used as a model in ecotoxicology because of its potential for comparative metabolomics research and for analysing the toxicity of bioactive substances in aquatic ecosystems [116,117]. The results showed that metribuzin is more hazardous than glyphosate and that its metabolome underwent commensurate modifications, according to metabolomics. Furthermore, pesticides significantly altered the amino acid pool in plants. According to the findings, electro-proteolytic activity was activated. In addition, the mixture of both herbicides activated the salicylate-signalling pathways [69]. Gamma-aminobutyric acid (GABA), salicylate, caffeine, a-trehalose, and squalene were some of the identified metabolites that were found to be biomarkers. These compounds have numerous functions in plant metabolism, including signalling, antioxidant defence, and structural protection [69].

#### 6.2.4. Gas Chromatography–Time-of-Flight Mass Spectrometry (GC–TOF MS)-Based Metabolomics

Plant metabolites that are over- or under-expressed due to environmental changes can be quickly screened using untargeted metabolomics-based GC–TOF MS. In addition, metabolomics based on GC–TOF MS provides the preliminary data that helps in understanding further mechanisms [118]. This technique produces a large number of metabolites that are adversely impacted by a given environmental stressor. A total of 150 metabolites were identified from vegetable samples based on their mass spectral (MS) signatures and retention index matches from up to 357 different metabolites that were occasionally found using GC–TOF MS. Among the identified metabolites, 30 were found to be altered [119]. In a similar study, Zhao et al. [120] used chromatography–time-of-flight mass spectrometry (GC–TOF MS)-based metabolomics to identify chemical alterations in *Cucumis sativus* (L.) plants treated with varied ecologically relevant concentrations of Cu-based fungicide until full maturity. Studies on metabolomics revealed that Cu fungicide disrupted Fe (iron) uptake in leaves. In addition, the quantum of macro nutrients such as calcium, phosphorous, sulphur, potassium, magnesium, and zinc drastically decreased under fungicidal stress. The chemical profile in cucumber fruits was noticeably changed as a result of fungicidal exposure, which was determined using metabolomics and partial least-squares discriminant analysis (PLS-DA). In response to stress exposure, a variety of metabolites up-regulated or down-regulated. Numerous C- and N-related pathways were altered, particularly the TCA cycle and galactose metabolism, demonstrating that applied C-based fungicide disrupted C and N metabolism.

Wu et al. [119] analysed the leaf metabolomic profiling of phosphite (Pi)-treated *Solanum tuberosum* L. (potato), and changes in metabolite pools were observed. Chlorogenic acid, caffeic acid, and salicylic acid, phytochemicals that play an important role in plant defence against biotic and abiotic stressors, increased following Pi treatments, supporting an efficient, indirect method of action. However, because there is no systematic evaluation of the recovery of metabolites during extraction or calibration of GC–TOF MS instruments, the untargeted metabolomics study only offers semi-quantitative data on changes in metabolite levels. As a result, it is necessary to quantify the changes using accurate quantification techniques.

#### 6.2.5. Combined NMR- and MS-Based Metabolomics

Due to the complimentary analytical benefits of both techniques, NMR- and MS-based metabolomics methodologies have been effectively combined in several investigations [121,122]. For instance, ^1^H NMR and GC–MS-based metabolomics studies have been performed to assess the effect of nano-copper pesticides on the nutritional content of *Cucumis sativus* (L.) fruits. Both platforms’ supervised partial least-squares discriminant analysis results revealed that samples of *C. sativus* fruit extracts were firmly categorised according to the amount of pollutant in the soil system. This suggests that contaminant exposure had an impact on the profile of fruit metabolites. According to GC–MS data, pollutant could either raise or reduce the content of certain sugars, organic acids, amino acids, and fatty acids. Additionally, only trigonelline, methyl nicotinamide, quinolinate, and imidazole metabolites were found and measured using ^1^H-NMR [120]. The combined use of the two platforms enabled researchers to understand how exposure to pollutants could affect the metabolite (nutrient supply) variations in *C. sativus* fruits. In another study using ^1^H NMR and GC–MS-based metabolomics, the response of nutrient uptake in *C. sativus* (L.) plants to various doses of other pollutants was assessed by Zhao et al. [118]. Moreover, ICP–MS revealed changes in the metabolism of mineral nutrients due to pollutants. The outcomes demonstrated that the absorption of both macro- and micro-nutrients (Na, P, S, Mo, Zn, and Fe) was hampered under stress. According to metabolomics data, root and leaf metabolites were significantly altered under stressed conditions. A defensive mechanism against stress was active, as evidenced through metabolic alterations in root exudate, up-regulation of amino acids, down-regulation of citric acid, up-regulation of ascorbic acid, and up-regulation of phenolic compounds. Therefore, these findings show that non-targeted ^1^H NMR and GC–MS-based metabolomics may be helpful in effectively pinpointing the physiological reactions occurring in plants while growing under abiotic stresses, including pesticides stress.

#### 6.2.6. Ultra-High-Performance Liquid Chromatography (UHPLC)-Based Metabolomics

In order to analyse the metabolome of a wide variety of crop plants, UHPLC-based metabolomics and other combination techniques have proven crucial in metabolomics research. In a study, Zhang et al. [123] used UHPLC to detect metabolic changes in *Lactuca sativa* (L.) leaves exposed to two insecticides, imidacloprid (IMD) and fenvalerate (FVE). Of these, FVE significantly reduced the leaf flavonoid (FVD) content, while IMD did not cause any significant changes in PPO and FVD. Furthermore, both insecticides altered root and shoot metabolism. A drastic increase in the level of amino acid metabolism but a remarkable decrease in carbohydrates metabolism was recorded following exposure to IMD. Furthermore, following pesticide application, the relative abundance of most organic acids and polyphenolic substances was considerably decreased.

#### 6.2.7. Ultra-High-Performance Liquid Chromatography Coupled with High-Resolution Mass Spectrometry (UHPLC–HRMS)

Based on high-resolution mass spectrometry (HRMS) and ultra-high-performance liquid chromatography (UHPLC), this metabolomics-based technique can identify small molecules. This analytical method has undergone complete validation in accordance with ISO17025 and WADA criteria, and the applicability of this method has been evaluated using real samples. Pan et al. [82] analysed the metabolomics profiling of pesticide-treated *C. sativus* (L.) leaves using the UHPLC-Q-Orbitrap–HRMS technique. It was observed that in fruits, antioxidants alleviated pesticide stress by up-regulating the shikimate–phenylpropanoid pathway by 1.3 times. Pesticide administration, however, enhanced both the tricarboxylic acid cycle (TCA) by 1.1-fold and the concentration of ROS-processing enzymes. These findings suggest that foliar pesticidal applications can control both associated metabolites and metabolic pathways, enhance cucumber fruit quality and antioxidant capability, and aid in pesticide detoxification.

#### 6.2.8. Matrix-Assisted Laser Desorption/Ionization Time-of-Flight Mass Spectrometry (MALDI–TOF MS)-Based Metabolomics

Before harvesting, edible plant components may have trace levels of insecticides and their by-products. Specialized methods with low inputs but greater sensitivity is required for the analysis of such substances. Having the ability to detect target molecules in real time might also be very advantageous (Table 3). Finding possible biomarkers is made simpler using UHPLC-QTOF/MS, which can evaluate samples’ lower amounts of distinct metabolites more quickly and thoroughly [124]. To date, various plant species exposed to environmental pollutants/contaminants have been studied using this technique. For instance, LC–MS and MALDI–TOF MS-based metabolomics analysis of chlorpyrifos-treated pods and beans of the *Phaseolus vulgaris* L. (common bean) plant has been performed by Fernandes et al. [125]. Pesticide application significantly decreased leaf pigments, and considerable reduction in triacyclglycerols revealed the alteration in lipidomic profiling of seeds and pods. In order to investigate the impact of the herbicides paraquat (PQT) and glyphosate (GP) on metabolomic profiling and protein expression, levels of rice leaves were studied using 2D gel electrophoresis combined with matrix-assisted laser desorption/ionization–time-of-flight (MALDI–TOF) mass spectrometry (MS) tools [126]. Here, from herbicide-treated leaf tissues, they identified 25 up-regulated/down-regulated variably expressed proteins. The effects of both herbicides were clearly seen to drastically reduce the major subunit of Rubisco. The treatments may cause oxidative stress in plants, as evidenced by the increased concentration of antioxidant enzymes in GP- and PQT-exposed samples. In another study, amiprophos methyl (APM), an herbicide that inhibits microtubule formation, was studied for its impact on microtubule and proteome activity in the mesocotyls, roots, and leaves of *Zea mays* (L.) using MALDI–TOF MS metabolomic tools [127]. In mesocotyls, roots, and leaves, the examination of 28 protein spots—15 new protein spots and 13 pre-existing proteins—had disappeared, which was observed under proteomic analysis. Furthermore, MALDI–TOF MS analysis of 10 protein spots revealed the presence of cold acclimation protein WCOR615, ubiquitin, maturase K, a ubiquitin-like protein, ferrdoxins, 2,4 Dienoyl-CoA reductases in the root, ATP-dependent protease, and retrotransposons in the leaves. These proteins were involved in a variety of biological processes, including protein synthesis. These findings imply that APM is a particular herbicide that disrupts microtubule dynamics and affects the expression of a number of proteins in crops, which may serve as potential biochemical markers to assess the herbicide’s toxicity in plants. Likewise, Fang et al. [128] applied the herbicide bentazon to rice plants in order to assess the expression and binding patterns of protein. They used 2D-DIGE coupled with MALDI–TOF MS/MS to investigate the bentazon-exposed leaf proteome in order to develop a thorough, pathway-oriented, mechanistic knowledge of the effects directly generated by the chemical herbicide. Results showed that proteins were stimulated by bentazon to change their relative levels of expression. Additionally, bentazon predominantly inhibited photosynthetic processes, and it had negative effects on carbohydrate metabolism and ATP generation. However, it also induced a number of stress-response proteins. In an experiment, Gholipour et al. [129] determined pesticidal residues accumulated on the fruit surface of *Solanum lycopersicum* (L.) by employing UV-MALDI–TOF MS technology. Fruits produced hydroponically in a greenhouse condition were treated one week later with a mixture of four pesticides and assessed for accumulation of pesticides on the fruit surface.

#### 6.2.9. Ultra-Performance Liquid Chromatography–Ion Mobility Spectroscopy–Quadrupole Time-of-Flight Mass Spectrometry (UPLC–IMS–QtoF)-Based Metabolomics

To understand the impact of the fungicide tebuconazole on lipid profiling and metabolites, Zhao et al. [130] conducted targeted and non-targeted metabolomics/lipidomics experiments using UPLC–QtoF MS and UPLC MS/MS. They observed that tebuconazole underwent a minimal amount of enantioselective degradation, and six potential degradation products were found. Tebuconazole exposure had a strong enantioselective impact on the endogenous metabolites involved in the metabolism of lipids, amino acids, nucleic acids, phenylpropanes, and flavonoids, as well as those involved in the metabolism of carbohydrates, amino acids, and vitamins. The activation of tebuconazole greatly increased nucleotide metabolism and the network of metabolic processes for nicotinic acid. Overall, it was discovered that tebuconazole exposure had a considerable effect on lipid and plant metabolism in lettuce. In a similar study, Lacina et al. [131] used a UHPLC–TOF MS-based metabolomic approach to investigate the comprehensive effects of 212 pesticide residues in QuEChERS extracts obtained from four plant matrices. Very recently, Wang et al. [132] employed UHPLC–QtoF MS to detect the metabolites in 10 different varieties of cooked, processed, and fruit radishes. Among the identified metabolites, three groups of metabolites were varied in composition, as shown through multivariate analysis, and a comparative analysis revealed that the metabolites that significantly differed in accumulation were primarily amino acids and their derivatives, lipids, flavonoids, hydroxycinnamate derivatives, and carbohydrates. Further, the UHPLC–QtoF MS metabolomic analysis revealed that radish fruit had higher amounts of metabolites, especially flavonoids. In order to identify and characterise residual pesticide metabolites in *Brassica* species, Bauer et al. [133] performed a metabolomic-based experiment using a UPLC–TWIMS–QtoF MS technique. The metabolomics revealed distinct degradation mechanisms and distribution profiles, including those for three difenoconazole metabolites, eight thiacloprid metabolites, and eleven azoxystrobin metabolites. In several plant organs, including leaves, stems, (broccoli) heads, and roots, various phase I and phase II metabolites of the pesticides have been found.

**Table 2:** Metabolomics approaches involved in the assessment of pesticide–plant interactions.

Pesticide Used	Chemical Class/Family	Dose Rate	Crop/Vegetable Used	Organ Involved	Way of Analyses/ Platform	Observation	Changes Observed	References
Mancozeb	Dithiocarbamate family	2.0 mg/L	*Lectuca sativa* (Lettuce)	Leaves	NMR-HRMAS	Negatively affected	➢ Variations in phenylalanine (PPA) and polyphenols (PPO) showed increased oxidative stress induced by herbicide. ➢ Herbicide exposure led to alterations in amino acids leading to up-regulation in the Krebs cycle. ➢ Abnormalities in sucrose, phospholipid, nucleotides, and nicotinamide metabolism were also observed.	[33]
Imidacloprid (IMD) and fenvalerate (FVE)	IMD; neonicotinoids, FVE; pyrethroid	10 mg/L	*Lactuca sativa* L. (lettuce)	Leaves	UHPLC	Negative effect	➢ Exposure of fenvalerate caused significant reduction (25%) in leaf flavonoid content (FVD). ➢ Imidacloprid did not cause any significant changes in PPO and FVD. ➢ Both insecticides significantly changed the metabolism in plant organs (roots and shoots) of lettuce. ➢ Imidacloprid action caused a drastic increase in metabolism of several amino acids; however, a remarkable reduction in carbohydrate metabolism was recorded. ➢ Furthermore, following pesticide exposure, the relative abundance of the majority of organic acids and polyphenolic substances drastically decreased.	[123]
Chlorpyrifos	Chlorinated organophosphate	0.576, 0.720, and 1.080 kg a. i./ha	*Oryza sativa* L (rice)	Leaves	GC-MS	Negatively affected	➢ Dramatic chlorpyrifos-induced reduction (60%) in six amino acid metabolism pathways and key DEG (mainly enriched in aspartate and glutamate metabolism) pathways were observed. ➢ Degradation of soluble proteins (down to 48.72% of the control).	[134]
Isoprocarb, carbofuran, and carbaryl	Carbamates	5.0 μg mL^−1^	*Brassica campestris* L. ssp. *Chinensis* (mustard) and *Makino* var. *communis*	Leaves	SPME	Negatively affected	➢ When exposed to carbamates, the fold changes of these important metabolites reduced from 0.78–1.07 to 0.28–0.82, demonstrating significant time-dependent dysregulations in the glucosinolate-related metabolite content.	[135]
Chlorpyrifos	Chlorinated organophosphate	0.02%, 0.06%, and 0.08%	*Phaseolus vulgaris* L. (Common bean)	Pod and beans	LC–MS and MALDI–TOF MS	Negatively affected	➢ Decreased leaf pigments and lipids in leaves and a considerable inhibition in triacyclglycerols was determined in seeds as well as pods.	[125]
Acetamiprid (ACE) and cyromazine (CYR)	ACD; chloropyridinyl neonicotinoids, CYMZ; aminotriazines	540 g a.i. ha 150 g a.i. ha	*Vigna unguiculata* L. (cowpea)	Leaves	LC–MS/MS	Negatively affected	➢ Both pesticides significantly affected amino acid metabolism, while CYR altered the flavonoid and sugar synthesis pathways. ➢ Alanine, glutamic acid, isoleucine, and phenylalanine levels and expression of genes associated to amino acids were lowered by ACE and CYR and elevated by MIX, respectively, in cowpea. ➢ Additionally, pesticide exposure to cowpea lowered the saccharide level and associated genes. ➢ Plants exposed to ACE had higher anthocyanin levels and lower levels of quercetin and naringenin chalcone.	[105]
Nano copper pesticides		400–800 mg/kg	*Cucumis sativus* L. (cucumber)		LC–MS/MS		➢ When other antioxidants were depleted, levels of benzoic acid, gallic acid hydrate, and p-coumaric acid increased, suggesting the activation of the defence mechanism.	[118]
Glyphosate (GP) and metribuzin (MBN)	GP; organophosphorus, MBN; triazinones	0.5, 1.0, 5.0, 10, 25 and 50 ppm	*Lemna minor* L.	Leaves	GC/EI/MS	Negative effect	➢ Both of the pesticides significantly altered the amino acid pool in plants, leading to increased concentrations of most known amino acids. ➢ As a result of the toxicity brought on by the pesticide mixture, salicylate-signalling pathways were also observed to be activated.	[69]
Lindane (HCH) and chlordecone (CLD)	LCH; organochlorine, CLD; organochlorine	2.5 µM to 25 µM	*Zea mays* L. (maize)	Root tips	^1^H-HR-MAS NMR	Negative effect	➢ Pesticide exposure increased the amount of fatty acids (FA) present, which was accompanied by a significant rise in oxidised FA. ➢ Under pesticide stress, an increase in LOX3 transcription levels was accompanied by a build-up of asparagine and oxidised fatty acids, which induced protein and lipid catabolism.	[70]
Fungicides	-	-	*Solanum tuberosum* L. (potato)	Tubers	UPLC–IMS–QtoF	Negative effect	➢ Metabolome of the tubers underwent substantial alterations following fungicide treatment. ➢ When a fungal or viral infection was imminent, flavonoids began to naturally increase in expression in the untreated group, whereas phytoalexin rishitinol was significantly more prevalent in the groups that received fungicide treatment.	[77]
Butachlor (BUTA), chlorpyrifos (CPF), tricyclazole (TZL)	BUTA; acetanilide class, CPF; organophosphates, TZL; triazolobenzothiazoles	CPF = 0.576, 0.720 and 1.080 kg a.i./ha. BUT = 0.90 and 1.574, 2.624 kg a.i./h.	*Oryza sativa* L. (rice)	Leaves	GC-MS	Negative effect	➢ The distribution of starch sucrose was disrupted by butachlor because it primarily affected five pathways for the metabolism of carbohydrates (38.5%), and more than 48.0% of differentially expressed genes (DEGs) were involved in these routes, as well as photosynthesis. ➢ Chlorpyrifos significantly inhibited the amino acid metabolism and DEG pathways. These effects resulted in an increase in free amino acid contents (up to 29.02% of the control) and the breakdown of soluble proteins. ➢ Fas metabolic pathways were considerably altered by tricyclazole (53.9%), and DEGs that largely code for oil–body membrane proteins. As a result, saturated Fas (palmitic acid and stearic acid) decreased and unsaturated Fas (linolenic and octadecadienoic acids) increased.	[78]
Chlorpyrifos	Organophosphate	2.0, 5.0 and 20.0 mg L^−1^	*Oryza sativa* L. (Rice)	Leaves and roots	LC–QTOF/MS	Negatively affected	➢ Higher concentration caused oxidative stress and inhibited the synthesis of chlorophyll and proteins. ➢ Affected the metabolic profiling of roots. ➢ Metabolism of glutamate amino acids, lipids, and flavonoids was significantly disrupted. ➢ A 1.32 to 2.19-fold change in quinic, aminobenzoic, and phosphoenolpyruvic acids was detected.	[136]
Thiamathoxam	Neonicotinoid	500 mgL^−1^	*Camellia sinensis* L.	Leaves	GC–MS and HPLC	Negatively affected	➢ Under insecticide stress, 113 metabolites were up-regulated, while 122 were found to be down-regulated. ➢ Following insecticide exposure, metabolic pathways of alanine, aspartate, and glutamate were severely affected.	[137]
Gulfosinate	-	1, 5, 10, and 15% *v*/*v*	*Stenotaphrum secundatum L.*	Plant	GC–MS	Negatively affected	➢ Caused a significant alteration in amino acid metabolism through up-regulation of isoleucine and phenylalanine.	[138]
Imazamox	Imidazolinone	0.036, 0.035, and 0.203 mg/L	*Lemna minor* L.	Leaves	LC–MS	Negatively affected	➢ Pesticide exposure caused the inhibition in metabolic pathways involved in pentose phosphate, photosynthesis, zeatin, and porphyrin. ➢ Additionally, phenylalanine metabolism, phenylpropanoid biosynthesis, zeatin biosynthesis, and secondary metabolite biosynthesis pathways were drastically influenced.	[117]
Perfluorooctanesulfonic acid	-	0, 25, and 50 mg/kg	*Triticum aestivum* L.	Roots and grains	HPLC/MS/MS	Negatively affected	➢ Higher concentration (50 mg/kg) reduced the chlorophyll content (49%) and root biomass (37%). ➢ The grain quality declined and the content of macro-elements such as P, K, and Mg was hampered. ➢ Sugar metabolites (e.g., sucrose, glucose 6-phosphate, fructose 6-phosphate, and trehalose) and PFOS also decreased the abundance of non-polar proteinogenic amino acids but increased the levels of polar amino acids in grains.	[139]

NMR–HRMAS = nuclear magnetic resonance and proton high-resolution magic angle-spinning; UHPLC = ultra-high-performance liquid chromatography, GC–MS = gas chromatography–mass spectroscopy; SPME = solid-phase microextraction; MALDI–TOF MS = matrix-assisted laser desorption/ionization time-of-flight mass spectroscopy; LC–MS = liquid chromatography mass spectroscopy.

**Table 3:** Advantages and limitations of NMR- and MS-based metabolomics analysis.

	NMR	MS
Sensitivity	Low but can be enhanced using cryo- and microprobes, dynamic nuclear polarisation, and greater field strengths.	High with a nanomolar detection threshold
Selectivity	Despite the fact that there are only a few selective experiments available, such as selective TOCSY, it is typically employed for nonselective analysis.	Can be used for both targeted and untargeted (selected and non-targeted) studies
Sample measurement	One measurement allows for the detection of all metabolites with an NMR concentration level.	For various kinds of metabolites, different chromatographic methods are typically required.
Sample recovery	Numerous analyses can be performed on the same sample without causing any damage; the sample can be recovered and kept for a long time.	Destructive method, but requires a small sample size
Reproducibility	Very high	Moderate
Number of detectable metabolites	30–100	300–1000+ (depending on whether GC–MS or LC–MS is used)
Sample preparation	Little sample preparation is required	More difficult; requires different columns and ionisation condition optimization
Tissue samples	Yes, using HR-MAS NMR tissue samples analysed directly	No, requires tissue extraction
Target analysis	Inapplicable to targeted analysis	More effective for specialised analysis
Sample analysis time	Quick—one measurement can be used to analyse the entire sample.	Longer and uses various chromatography methods depending on the metabolites being examined
In vivo studies	Yes—widely used for ^1^H magnetic resonance spectroscopy (and to a lesser degree ^31^P and ^13^C)	No—although desorption electrospray ionization (DESI) may be a useful way to sample tissues in a minimally invasive way during surgery
Instrument cost	More expensive and occupies more space	More affordable (cheaper) and compact
Sample cost	Low cost per sample	High cost per sample

Adapted and modified from Emwas [140].

## 7. Challenges in Metabolomics and Future Prospects

Metabolomics can be used in environmental toxicology to study how various crops are affected by chemical pesticides. For instance, the application of lipidomics, which reveals modifications in lipid composition of plant tissues following exposure to pesticides, has a significant potential to uncover the mode of action, illuminate toxicity mechanisms, and expose exposure pathways [141]. In addition to the capability of high-throughput metabolite analysis, metabolomics also provides information regarding potential toxicity processes. Environmental metabolomics has made it easier over the past ten years to identify the effects of harmful contaminants such as xenobiotics and other related substances. A deeper understanding of molecular mechanisms of action in (eco)toxicological research will be greatly aided by the extensive computational models that may be created through the analysis of thousands of metabolites at the cellular level. 

Comprehensive investigation of all metabolites impacted by various stressors still presents a difficulty because of the physiochemical diversity of the complete metabolome. Early initiatives in metabolomics used solitary systems for analysis. Recent studies have, however, benefited from combined analysis platforms, which integrate various analytical methodologies to increase the coverage of metabolites, thanks to developments in instrumentation and analysis. The identification of metabolites is a significant obstacle for untargeted metabolomics. An additional difficulty in metabolomics is choosing the right LC columns to effectively separate extremely polar metabolites that are poorly maintained in traditional reversed-phase columns without losing the separation of less polar metabolites. However, combining revolutionary mixed-mode column technologies with UHPLC may increase the coverage of metabolome change detection. We anticipate that such advancements will considerably aid environmental science in the future, allowing for the discovery of biomarkers for early exposure or elucidating an organismal state. 

Currently, the metabolomic scientific community is trying to further the application of metabolomics and transcriptomics within the framework of the OECD’s programme for chemical safety. Furthermore, usage of NAMs, computational pipeline development, and ongoing mass spectrometry advancements will make it easier to meet the requirements necessary for laboratory-based toxicological investigations in plant science. Additionally, metabolomics (and transcriptomics) will play a significant role in toxicology laboratories in the near future to evaluate the effects of pollutants, including chemical pesticides, at a molecular level, providing a wealth of useful information to ecotoxicological studies, assuming the use of omics technologies for chemical safety regulation is approved.

## Abbreviations

GC–MS	Gas chromatography–mass spectrophotometry
LC–MS	Liquid chromatography–mass spectrophotometry
HPLC	High-performance liquid chromatography
UPLC–IMS–QtoF	Ultra-performance liquid chromatography–ion mobility spectroscopy–quadrupole time-of-flight mass spectrometry
MALDI–TOF MS	Matrix-assisted laser desorption/ionization time-of-flight mass spectrometry
GC/EI/MS	Gas chromatography/electron impact mass spectrometry
^1^H-HR-MAS NMR	High-resolution magic angle-spinning nuclear magnetic resonance
ROS	Reactive oxygen species
H_2_O_2_	Hydrogen peroxide
(O^−2^)	Superoxide anions
RBOH1	Respiratory burst oxidase homologous 1
NAD	Nicotinamide adenine dinucleotide
NADP	Nicotinamide adenine dinucleotide phosphate
ATP	Adenosine triphosphate
(ΔΨm)	Mitochondrial membrane potential
DNA	Deoxyribonucleic acid
TCA	Tricarboxylic acid cycle
GC-LRMS	Gas chromatography coupled to low-resolution mass spectrometry
NMR	Nuclear magnetic resonance
DI–FTICR–MS	Direct-infusion Fourier-transform ion cyclotron-resonance
DMSO	Dimethyl sulfoxide
HMDS	Hexamethyldisilazane
MTBSTFA	N-tert-butyldimethylsilyl-N-methyltrifluoroacetamide
FTICR–MS	Fourier-transform ion cyclotron resonance (FTICR) mass spectrometry
GC–TOF MS	Gas chromatography (GC) coupled to time-of-flight mass spectrometry
ICP–MS	Inductively coupled plasma mass spectrometry
UHPLC	Ultra-high-performance liquid chromatography
2D-DIGE	Two-dimensional difference gel electrophoresis
QuEChERS	Quick, easy, cheap, effective, rugged, and safe
MS (UPLC–TWIMS–QTOF)	Ultra-performance liquid chromatography system coupled to a high-resolution quadrupole/traveling wave ion mobility spectrometry/time-of-flight

## References

[1] USEPA—United States of Environmental Protection Agengy About Pesticides. U.S. EPA. https://www.epa.gov/pesticide-registration/about-pesticide-registration.

[2] Mesnage R., Székács A., Zaller J.G. (2021). Herbicides: Brief history, agricultural use, and potential alternatives for weed control. Herbicides.

[3] Wojciechowska M., Stepnowski P., Gołębiowski M. (2016). The use of insecticides to control insect pests. Invertebr. Surviv. J..

[4] Singh S., Kumar V., Dhanjal D.S., Singh J. (2020). Herbicides and plant growth regulators: Current developments and future challenges. Natural Bioactive Products in Sustainable Agriculture.

[5] Price C.L., Parker J.E., Warrilow A.G., Kelly D.E., Kelly S.L. (2015). Azole fungicides–understanding resistance mechanisms in agricultural fungal pathogens. Pest Manag. Sci..

[6] Shahid M., Khan M.S. (2022). Ecotoxicological implications of residual pesticides to beneficial soil bacteria: A review. Pestic. Biochem. Physiol..

[7] Yadav I.C., Devi N.L., Syed J.H., Cheng Z., Li J., Zhang G., Jones K.C. (2015). Current status of persistent organic pesticides residues in air, water, and soil, and their possible effect on neighboring countries: A comprehensive review of India. Sci. Total Environ..

[8] Shahid M., Khan M.S., Ahmed B., Syed A., Bahkali A.H. (2021). Physiological disruption, structural deformation and low grain yield induced by neonicotinoid insecticides in chickpea: A long term phytotoxicity investigation. Chemosphere.

[9] Shahid M., Khan M.S., Syed A., Marraiki N., Elgorban A.M. (2021). *Mesorhizobium ciceri* as biological tool for improving physiological, biochemical and antioxidant state of *Cicer aritienum* (L.) under fungicide stress. Sci. Rep..

[10] Shahid M., Manoharadas S., Chakdar H., Alrefaei A.F., Albeshr M.F., Almutairi M.H. (2021). Biological toxicity assessment of carbamate pesticides using bacterial and plant bioassays: An *in-vitro* approach. Chemosphere.

[11] Khan S., Shahid M., Khan M.S., Syed A., Bahkali A.H., Elgorban A.M., Pichtel J. (2020). Fungicide-tolerant plant growth-promoting rhizobacteria mitigate physiological disruption of white radish caused by fungicides used in the field cultivation. Int. J. Environ. Res. Public Health.

[12] Shahid M., Khan M.S., Kumar M. (2019). Kitazin-pea interaction: Understanding the fungicide induced nodule alteration, cytotoxicity, oxidative damage and toxicity alleviation by *Rhizobium leguminosarum*. RSC Adv..

[13] Shahid M., Khan M.S. (2019). Fungicide tolerant *Bradyrhizobium japonicum* mitigate toxicity and enhance greengram production under hexaconazole stress. J. Environ. Sci..

[14] Shahid M., Ahmed B., Zaidi A., Khan M.S. (2018). Toxicity of fungicides to *Pisum sativum*: A study of oxidative damage, growth suppression, cellular death and morpho-anatomical changes. RSC Adv..

[15] Peng X., Wang N., Sun S., Geng L., Guo N., Liu A., Chen S., Ahammed G.J. (2023). Reactive oxygen species signalling is involved in melatonin-induced reduction of chlorothalonil residue in tomato leaves. J. Hazard. Mater..

[16] Lydon J., Duke S.O. (1989). Pesticide effects on secondary metabolism of higher plants. Pestic. Sci..

[17] Li X., Li Y., Ahammed G.J., Zhang X.N., Ying L., Zhang L., Yan P., Zhang L.P., Li Q.Y., Han W.Y. (2019). RBOH1-dependent apoplastic H_2_O_2_ mediates epigallocatechin-3-gallate-induced abiotic stress tolerance in *Solanum lycopersicum* L.. Environ. Exp. Bot..

[18] Shahid M., Khan M. (2018). Glyphosate induced toxicity to chickpea plants and stress alleviation by herbicide tolerant phosphate solubilizing *Burkholderia cepacia* PSBB1 carrying multifarious plant growth promoting activities. 3 Biotech.

[19] Xiao Y.M., Esser L., Zhou F., Li C., Zhou Y.H., Yu C.A., Qin Z.H., Xia D. (2014). Studies on inhibition of respiratory cytochrome bc 1 complex by the fungicide pyrimorph suggest a novel inhibitory mechanism. PLoS ONE.

[20] Shahid M., Khan M.S., Zaidi A. (2020). Fungicide toxicity to legumes and its microbial remediation: A current perspective. Pestic. Crop. Prod. Physiol. Biochem. Action.

[21] Sharma I., Bhardwaj R., Pati P.K. (2015). Exogenous application of 28-homobrassinolide modulates the dynamics of salt and pesticides induced stress responses in an elite rice variety Pusa Basmati-1. J. Plant Growth Reg..

[22] Shakir S.K., Irfan S., Akhtar B., Daud M.K., Taimur N., Azizullah A. (2018). Pesticide-induced oxidative stress and antioxidant responses in tomato (*Solanum lycopersicum*) seedlings. Ecotoxicology.

[23] Chen S., Yin C., Strasser R.J., Yang C., Qiang S. (2012). Reactive oxygen species from chloroplasts contribute to 3-acetyl-5-isopropyltetramic acid-induced leaf necrosis of *Arabidopsis thaliana*. Plant Physiol. Biochem..

[24] Stanley J., Preetha G., Stanley J. (2016). Pesticide Toxicity to Non-Target Organisms.

[25] Hall R.D. (2006). Plant metabolomics: From holistic hope, to hype, to hot topic. New Phytol..

[26] Rinschen M.M., Ivanisevic J., Giera M., Siuzdak G. (2019). Identification of bioactive metabolites using activity metabolomics. Nat. Rev. Mol. Cell Biol..

[27] Allwood J.W., De Vos R.C., Moing A., Deborde C., Erban A., Kopka J., Goodacre R., Hall R.D. (2011). Plant metabolomics and its potential for systems biology research: Background concepts, technology, and methodology. Methods Enzymol..

[28] Wishart D.S. (2019). Metabolomics for investigating physiological and pathophysiological processes. Physiol. Rev..

[29] Viant M.R. (2008). Recent developments in environmental metabolomics. Mol. Biosyst..

[30] Matich E.K., Soria N.G.C., Aga D.S., Atilla-Gokcumen G.E. (2019). Applications of metabolomics in assessing ecological effects of emerging contaminants and pollutants on plants. J. Hazard. Mater..

[31] Dreher K. (2014). Putting the plant metabolic network pathway databases to work: Going offline to gain new capabilities. Plant Metabolism.

[32] Kanehisa M., Goto S. (2000). KEGG: Kyoto Encyclopedia of Genes and Genomes. Nucleic Acids Res..

[33] Pereira S.I., Figueiredo P.I., Barros A.S., Dias M.C., Santos C., Duarte I.F., Gil A.M. (2014). Changes in the metabolome of lettuce leaves due to exposure to mancozeb pesticide. Food Chem..

[34] Zhang H., Liu J., Wen R., Chen Q., Kong B. (2021). Metabolomics profiling reveals defense strategies of *Pediococcus pentosaceus* R1 isolated from Harbin dry sausages under oxidative stress. LWT.

[35] Zhao Y., Zhang L., Zhao C., Hu C., Li Y., Zhao J., Zhang J., Li L., Chang Y., Wang F. (2015). Metabolic responses of rice leaves and seeds under transgenic backcross breeding and pesticide stress by pseudo-targeted metabolomics. Metabolomics.

[36] Hu L., Liu J., Zhang W., Wang T., Zhang N., Lee Y.H., Lu H. (2020). Functional metabolomics decipher biochemical functions and associated mechanisms underlie small-molecule metabolism. Mass Spectrom. Rev..

[37] Labine L.M., Simpson M.J. (2020). The use of nuclear magnetic resonance (NMR) and mass spectrometry (MS)–based metabolomics in environmental exposure assessment. Curr. Opinion Environ. Sci. Health.

[38] Emwas A.H., Roy R., McKay R.T., Tenori L., Saccenti E., Gowda G.N., Raftery D., Alahmari F., Jaremko L., Jaremko M. (2019). NMR spectroscopy for metabolomics research. Metabolites.

[39] Bothwell J.H., Griffin J.L. (2011). An introduction to biological nuclear magnetic resonance spectroscopy. Biol. Rev..

[40] Shaykhutdinov R.A., MacInnis G.D., Dowlatabadi R., Weljie A.M., Vogel H.J. (2009). Quantitative analysis of metabolite concentrations in human urine samples using ^13^C {^1^H} NMR spectroscopy. Metabolomics.

[41] Traverso A., Wee L., Dekker A., Gillies R. (2018). Repeatability and reproducibility of radiomic features: A systematic review. Int. J. Radiation Oncol. Biol. Phys..

[42] Valentino G., Graziani V., D’Abrosca B., Pacifico S., Fiorentino A., Scognamiglio M. (2020). NMR-based plant metabolomics in nutraceutical research: An overview. Molecules.

[43] Finehout E.J., Lee K.H. (2004). An introduction to mass spectrometry applications in biological research. Biochem. Mol. Biol. Educ..

[44] Blakley C.R., Carmody J.J., Vestal M.L. (1980). Liquid chromatograph-mass spectrometer for analysis of non-volatile samples. Analytic. Chem..

[45] Zhao S., Li L. (2020). Chemical derivatization in LC-MS-based metabolomics study. Trends Analytic. Chem..

[46] Want E.J., Cravatt B.F., Siuzdak G. (2005). The expanding role of mass spectrometry in metabolite profiling and characterization. Chembiochem.

[47] Cajka T., Fiehn O. (2016). Toward merging untargeted and targeted methods in mass spectrometry-based metabolomics and lipidomics. Anal. Chem..

[48] Hill C.B., Roessner U. (2013). Metabolic profiling of plants by GC–MS. Handbook of Plant Metabolomics.

[49] Terracio L., Schwabe K.G. (1981). Freezing and drying of biological tissues for electron microscopy. J. Histochem. Cytochem..

[50] Miazek K., Kratky L., Sulc R., Jirout T., Aguedo M., Richel A., Goffin D. (2017). Effect of organic solvents on microalgae growth, metabolism and industrial bioproduct extraction: A review. Int. J. Mol. Sci..

[51] Joshi D.R., Adhikari N. (2019). An overview on common organic solvents and their toxicity. J. Pharm. Res. Int..

[52] Nelson W.M. (2003). Green Solvents for Chemistry: Perspectives and Practice.

[53] Nanda B., Sailaja M., Mohapatra P., Pradhan R.K., Nanda B.B. (2021). Green solvents: A suitable alternative for sustainable chemistry. Mater. Today Proc..

[54] Zalewska K.A.M. (2014). Development of Novel Ionic Liquids Based on Biological Molecules. Ph.D. Thesis.

[55] Yang Z., Pan W. (2005). Ionic liquids: Green solvents for nonaqueous bio catalysis. Enzym. Microb. Technol..

[56] Ramos L., Kristenson E.M., Brinkman U.T. (2002). Current use of pressurised liquid extraction and subcritical water extraction in environmental analysis. J. Chromatogr. A.

[57] Sapkale G.N., Patil S.M., Surwase U.S., Bhatbhage P.K. (2010). Supercritical fluid extraction. Int. J. Chem. Sci..

[58] Abbasi N.M., Farooq M.Q., Anderson J.L. (2021). Modulating solvation interactions of deep eutectic solvents formed by ammonium salts and carboxylic acids through varying the molar ratio of hydrogen bond donor and acceptor. J. Chromatogr. A.

[59] Lam S.M., Tian H., Shui G. (2017). Lipidomics, en route to accurate quantitation. Biochim. Biophys. Acta (BBA)-Mol. Cell Biol. Lipids.

[60] Salem M.A., Jüppner J., Bajdzienko K., Giavalisco P. (2016). Protocol: A fast, comprehensive and reproducible one-step extraction method for the rapid preparation of polar and semi-polar metabolites, lipids, proteins, starch and cell wall polymers from a single sample. Plant Methods.

[61] Corrêa P.S., Morais Júnior W.G., Martins A.A., Caetano N.S., Mata T.M. (2020). Microalgae biomolecules: Extraction, separation and purification methods. Processes.

[62] Lee J.H., Zhu J. (2021). Analyses of short-chain fatty acids and exhaled breath volatiles in dietary intervention trials for metabolic diseases. Exp. Biol. Med..

[63] Smart K.F., Aggio R.B., Van Houtte J.R., Villas-Bôas S.G. (2010). Analytical platform for metabolome analysis of microbial cells using methyl chloroformate derivatization followed by gas chromatography–mass spectrometry. Nat. Protoc..

[64] Maia M., Monteiro F., Sebastiana M., Marques A.P., Ferreira A.E., Freire A.P., Cordeiro C., Figueiredo A., Silva M.S. (2016). Metabolite extraction for high-throughput FTICR-MS-based metabolomics of grapevine leaves. EuPA Open Proteom..

[65] Brown S.A., Simpson A.J., Simpson M.J. (2008). Evaluation of sample preparation methods for nuclear magnetic resonance metabolic profiling studies with *Eisenia fetida*. Environ. Toxicol. Chem. Int. J..

[66] Guy G.R., Philip R., Tan Y.H. (1994). Analysis of cellular phosphoproteins by two-dimensional gel electrophoresis: Applications for cell signaling in normal and cancer cells. Electrophoresis.

[67] McKelvie J.R., Yuk J., Xu Y., Simpson A.J., Simpson M.J. (2009). ^1^H NMR and GC/MS metabolomics of earthworm responses to sub-lethal DDT and endosulfan exposure. Metabolomics.

[68] Liu N., An X., Wang Y., Qi J. (2023). Metabolomics Analysis Reveals the Effect of Fermentation to Secondary Metabolites of Chenopodium album L. Based on UHPLC-QQQ-MS. Fermentation.

[69] Kostopoulou S., Ntatsi G., Arapis G., Aliferis K.A. (2020). Assessment of the effects of metribuzin, glyphosate, and their mixtures on the metabolism of the model plant *Lemna minor* L. applying metabolomics. Chemosphere.

[70] Blondel C., Khelalfa F., Reynaud S., Fauvelle F., Raveton M. (2016). Effect of organochlorine pesticides exposure on the maize root metabolome assessed using high-resolution magic-angle spinning ^1^H-NMR spectroscopy. Environ. Pollut..

[71] Hui-Qin P.A.N., Heng Z.H.O.U., Shui M.I.A.O., De-An G.U.O., Zhang X.L., Qing H.U., Xiu-Hong M.A.O., Shen J.I. (2021). Plant metabolomics for studying the effect of two insecticides on comprehensive constituents of *Lonicerae Japonicae* Flos. Chin. J. Nat. Med..

[72] Assefa A.D., Hur O.S., Hahn B.S., Kim B., Ro N.Y., Rhee J.H. (2021). Nutritional metabolites of red pigmented lettuce (*Lactuca sativa*) germplasm and correlations with selected phenotypic characters. Foods.

[73] Sarry J.E., Kuhn L., Ducruix C., Lafaye A., Junot C., Hugouvieux V., Jourdain A., Bastien O., Fievet J.B., Vailhen D. (2006). The early responses of Arabidopsis thaliana cells to cadmium exposure explored by protein and metabolite profiling analyses. Proteomics.

[74] Zhou J., Wang J., Shi K., Xia X.J., Zhou Y.H., Yu J.Q. (2012). Hydrogen peroxide is involved in the cold acclimation-induced chilling tolerance of tomato plants. Plant Physiol. Biochem..

[75] Huang Y., Adeleye A.S., Zhao L., Minakova A.S., Anumol T., Keller A.A. (2019). Antioxidant response of cucumber (*Cucumis sativus*) exposed to nano copper pesticide: Quantitative determination via LC-MS/MS. Food Chem..

[76] Pretali L., Bernardo L., Butterfield T.S., Trevisan M., Lucini L. (2016). Botanical and biological pesticides elicit a similar induced systemic response in tomato (*Solanum lycopersicum*) secondary metabolism. Phytochemistry.

[77] Claassen C., Ebel E., Kuballa J., Rohn S. (2019). Impacts of fungicide treatment and conventional fertilization management on the potato metabolome (*Solanum tuberosum* L.) evaluated with UPLC-IMS-QToF. J. Agric Food Chem..

[78] Liu N., Zhu L. (2020). Metabolomic and transcriptomic investigation of metabolic perturbations in *Oryza sativa* L. triggered by three pesticides. Environ. Sci. Tech..

[79] Zhou J., Zhang L., Chang Y., Lu X., Zhu Z., Xu G. (2012). Alteration of leaf metabolism in Bt-transgenic rice (*Oryza sativa* L.) and its wild type under insecticide stress. J. Proteome Res..

[80] Siano G.G., Pérez I.S., García M.D.G., Galera M.M., Goicoechea H.C. (2011). Multivariate curve resolution modeling of liquid chromatography–mass spectrometry data in a comparative study of the different endogenous metabolites behavior in two tomato cultivars treated with carbofuran pesticide. Talanta.

[81] Zhao Q., Xi J., Xu D., Jin Y., Wu F., Tong Q., Yin Y., Xu X. (2022). A comparative HS-SPME/GC-MS-based metabolomics approach for discriminating selected japonica rice varieties from different regions of China in raw and cooked form. Food Chem..

[82] Pan L., Zhou C., Jing J., Zhuang M., Zhang J., Wang K., Zhang H. (2022). Metabolomics analysis of cucumber fruit in response to foliar fertilizer and pesticides using UHPLC-Q-Orbitrap-HRMS. Food Chem..

[83] Mahdavi V., Farimani M.M., Fathi F., Ghassempour A. (2015). A targeted metabolomics approach toward understanding metabolic variations in rice under pesticide stress. Anal. Biochem..

[84] Amoroso V.B., Mendez R.A., Junio H.A., Molino R.J.E.J., Pescadero I.R., Villalobos A.P. (2021). Characterization of a natural fungicide from an indigenous plant *Tasmannia piperita* (Hook. f.) Miers Extract: Stability, Metabolomics, and In silico Studies. Philipp. J. Sci..

[85] Das A.B., Goud V.V., Das C. (2017). Extraction of phenolic compounds and anthocyanin from black and purple rice bran (*Oryza sativa* L.) using ultrasound: A comparative analysis and phytochemical profiling. Indus. Crops Prod..

[86] Li Y., Zhang Q., Yu Y., Li X., Tan H. (2020). Integrated proteomics, metabolomics and physiological analyses for dissecting the toxic effects of halo-sulfuron-methyl on soybean seedlings (*Glycine max* merr.). Plant Physiol. Biochem..

[87] Kruger N.J., Troncoso-Ponce M.A., Ratcliffe R.G. (2008). ^1^H NMR metabolite fingerprinting and metabolomic analysis of perchloric acid extracts from plant tissues. Nat. Protoc..

[88] Lucarini M., Di Cocco M.E., Raguso V., Milanetti F., Durazzo A., Lombardi-Boccia G., Santini A., Delfini M., Sciubba F. (2020). NMR-based metabolomic comparison of *Brassica oleracea* (var. italica): Organic and conventional farming. Foods.

[89] Hurtado C., Parastar H., Matamoros V., Piña B., Tauler R., Bayona J.M. (2017). Linking the morphological and metabolomic response of *Lactuca sativa* L. exposed to emerging contaminants using GC× GC-MS and chemometric tools. Sci. Rep..

[90] Kazimierczak R., Hallmann E., Lipowski J., Drela N., Kowalik A., Püssa T., Matt D., Luik A., Gozdowski D., Rembiałkowska E. (2014). Beetroot (*Beta vulgaris* L.) and naturally fermented beetroot juices from organic and conventional production: Metabolomics, antioxidant levels and anticancer activity. J. Sci. Food Agric..

[91] Hoult D.I., Busby S.J.W., Gadian D.G., Radda G.K., Richards R.E., Seeley P.J. (1974). Observation of tissue metabolites using 31P nuclear magnetic resonance. Nature.

[92] Krishnan P., Kruger N.J., Ratcliffe R.G. (2005). Metabolite fingerprinting and profiling in plants using NMR. J. Exp. Bot..

[93] Crook A.A., Powers R. (2020). Quantitative NMR-based biomedical metabolomics: Current status and applications. Molecules.

[94] Moco S., Schneider B., Vervoort J. (2000). Plant micro metabolomics: The analysis of endogenous metabolites present in a plant cell or tissue. J. Proteome Res..

[95] Wu H., Southam A.D., Hines A., Viant M.R. (2008). High-throughput tissue extraction protocol for NMR-and MS-based metabolomics. Anal. Biochem..

[96] Garcia-Perez I., Posma J.M., Serrano-Contreras J.I., Boulangé C.L., Chan Q., Frost G., Stamler J., Elliott P., Lindon J.C., Holmes E. (2020). Identifying unknown metabolites using NMR-based metabolic profiling techniques. Nat. Protoc..

[97] Fraga-Corral M., Carpena M., Garcia-Oliveira P., Pereira A.G., Prieto M.A., Simal-Gandara J. (2022). Analytical metabolomics and applications in health, environmental and food science. Crit. Rev. Analytic. Chem..

[98] Khakimov B., Bak S., Engelsen S.B. (2014). High-throughput cereal metabolomics: Current analytical technologies, challenges and perspectives. J. Cereal Sci..

[99] Bauermeister A., Mannochio-Russo H., Costa-Lotufo L.V., Jarmusch A.K., Dorrestein P.C. (2022). Mass spectrometry-based metabolomics in microbiome investigations. Nat. Rev. Microbiol..

[100] Sahoo A.K., Au W.C., Yang C.S., Mai C.M., Pan C.L. THz Spectroscopy as Non-destructive Alternative to Secondary Ion Mass Spectroscopy. Proceedings of the 45th International Conference on Infrared, Millimeter, and Terahertz Waves (IRMMW-THz).

[101] Allen F., Pon A., Greiner R., Wishart D. (2016). Computational prediction of electron ionization mass spectra to assist in GC/MS compound identification. Anal. Chem..

[102] Vinaixa M., Schymanski E.L., Neumann S., Navarro M., Salek R.M., Yanes O. (2016). Mass spectral databases for LC/MS-and GC/MS-based metabolomics: State of the field and future prospects. Trends Analytic. Chem..

[103] Xiao J.F., Zhou B., Ressom H.W. (2012). Metabolite identification and quantitation in LC-MS/MS-based metabolomics. Trends Anal. Chem..

[104] Majchrzak T., Wojnowski W., Lubinska-Szczygeł M., Różańska A., Namieśnik J., Dymerski T. (2018). PTR-MS and GC-MS as complementary techniques for analysis of volatiles: A tutorial review. Anal. Chim. Acta.

[105] Zhang S., Yin F., Li J., Ren S., Liang X., Zhang Y., Wang L., Wang M., Zhang C. (2022). Transcriptomic and metabolomic investigation of metabolic disruption in *Vigna unguiculata* L. triggered by acetamiprid and cyromazine. Ecotoxicol. Environ. Safety.

[106] Theodoridis G., Gika H.G., Wilson I.D. (2008). LC-MS-based methodology for global metabolite profiling in metabonomics/metabolomics. Trends Analytic. Chem..

[107] Cook D.W., Rutan S.C., Stoll D.R., Carr P.W. (2015). Two-dimensional assisted liquid chromatography–a chemometric approach to improve accuracy and precision of quantitation in liquid chromatography using 2D separation, dual detectors, and multivariate curve resolution. Anal. Chim. Acta.

[108] Mondello L., Tranchida P.Q., Dugo P., Dugo G. (2008). Comprehensive two-dimensional gas chromatography-mass spectrometry: A review. Mass Spectrom. Rev..

[109] Mollerup C.B., Rasmussen B.S., Johansen S.S., Mardal M., Linnet K., Dalsgaard P.W. (2019). Retrospective analysis for valproate screening targets with liquid chromatography–high resolution mass spectrometry with positive electrospray ionization: An omics-based approach. Drug Test. Anal..

[110] Naz S., Gallart-Ayala H., Reinke S.N., Mathon C., Blankley R., Chaleckis R., Wheelock C.E. (2017). Development of a liquid chromatography–high resolution mass spectrometry metabolomics method with high specificity for metabolite identification using all ion fragmentation acquisition. Anal. Chem..

[111] Danek M., Plonka J., Barchanska H. (2021). Metabolic profiles and non-targeted LC–MS/MS approach as a complementary tool to targeted analysis in assessment of plant exposure to pesticides. Food Chem..

[112] Anagnostopoulos C., Stasinopoulou P., Kanatas P., Travlos I. (2020). Differences in metabolism of three Conyza species to herbicides glyphosate and triclopyr revealed by LC-MS/MS. Chil. J. Agric. Res..

[113] González-Torralva F., Rojano-Delgado A.M., de Castro M.D.L., Mülleder N., De Prado R. (2012). Two non-target mechanisms are involved in glyphosate-resistant horseweed (*Conyza canadensis* L. Cronq.) biotypes. J. Plant Physiol..

[114] Yu Y., Wang S., Zhang Q., Yang Y., Chen Y., Liu X., Feng C., Hu D., Lu P. (2019). Dissipation, residues, and risk assessment of imidacloprid in Zizania latifolia and purple sweet potato under field conditions using LC-MS/MS. J. Environ. Sci. Health Part B.

[115] Vu-Duc N., Nguyen-Quang T., Le-Minh T., Nguyen-Thi X., Tran T.M., Vu H.A., Nguyen L.A., Doan-Duy T., Van Hoi B., Vu C.T. (2019). Multiresidue pesticides analysis of vegetables in Vietnam by ultrahigh-performance liquid chromatography in combination with high-resolution mass spectrometry (UPLC-Orbitrap MS). J. Anal. Methods Chem..

[116] Lee H., Depuydt S., Shin K., Choi S., Kim G., Lee Y.H., Park J.T., Han T., Park J. (2021). Assessment of Various Toxicity Endpoints in Duckweed (*Lemna minor*) at the Physiological, Biochemical, and Molecular Levels as a Measure of Diuron Stress. Biology.

[117] Aliferis K.A., Materzok S., Paziotou G.N., Chrysayi-Tokousbalides M. (2009). *Lemna minor* L. as a model organism for ecotoxicological studies performing ^1^H NMR fingerprinting. Chemosphere.

[118] Zhao L., Huang Y., Zhou H., Adeleye A.S., Wang H., Ortiz C., Mazer S.J., Keller A.A. (2016). GC-TOF-MS based metabolomics and ICP-MS based metallomics of cucumber (*Cucumis sativus*) fruits reveal alteration of metabolites profile and biological pathway disruption induced by nano copper. Environ. Sci. Nano.

[119] Wu L., Gao X., Xia F., Joshi J., Borza T., Wang-Pruski G. (2019). Biostimulant and fungicidal effects of phosphite assessed by GC-TOF-MS analysis of potato leaf metabolome. Physiol. Mol. Plant Pathol..

[120] Zhao L., Hu J., Huang Y., Wang H., Adeleye A., Ortiz C., Keller A.A. (2017). ^1^H NMR and GC–MS based metabolomics reveal nano-Cu altered cucumber (*Cucumis sativus*) fruit nutritional supply. Plant Physiol. Biochem..

[121] Nagana Gowda G.A., Raftery D. (2017). Recent advances in NMR-based metabolomics. Anal. Chem..

[122] Zhang A., Sun H., Wang P., Han Y., Wang X. (2012). Modern analytical techniques in metabolomics analysis. Analyst.

[123] Zhang Y., Huang L., Liu L., Cao X., Sun C., Lin X. (2022). Metabolic disturbance in lettuce (*Lactuca sativa*) plants triggered by imidacloprid and fenvalerate. Sci. Total Environ..

[124] Gagnebin Y., Tonoli D., Lescuyer P., Ponte B., de Seigneux S., Martin P.Y., Schappler J., Boccard J., Rudaz S. (2017). Metabolomic analysis of urine samples by UHPLC-QTOF-MS: Impact of normalization strategies. Anal. Chim. Acta.

[125] Fernandes C., Figueira E., Tauler R., Bedia C. (2018). Exposure to chlorpyrifos induces morphometric, biochemical and lipidomic alterations in green beans (*Phaseolus vulgaris*). Ecotoxicol. Environ. Saf..

[126] Ahsan N., Lee D.G., Lee K.W., Alam I., Lee S.H., Bahk J.D., Lee B.H. (2008). Glyphosate-induced oxidative stress in rice leaves revealed by proteomic approach. Plant Physiol. Biochem..

[127] Wang Z., Li Q., Zhao J., Peng Y. (2011). Investigation of the effect of herbicide amiprophos methyl on spindle formation and proteome change in maize by immunofluorescence and proteomic technique. Cytologia.

[128] Fang Y., Lu H., Chen S., Zhu K., Song H., Qian H. (2015). Leaf proteome analysis provides insights into the molecular mechanisms of bentazon detoxification in rice. Pestic. Biochem. Physiol..

[129] Gholipour Y., Erra-Balsells R., Nonami H. (2012). Detection of pesticides on tomato fruit surface by ultraviolet matrix-assisted laser desorption/ionization mass spectrometry. Environ. Control Biol..

[130] Zhao L., Zhang Y., Wang L., Liu X., Zhang J., He Z. (2022). Stereoselective metabolomic and lipidomic responses of lettuce (*Lactuca sativa* L.) exposing to chiral triazole fungicide tebuconazole. Food Chem..

[131] Lacina O., Urbanova J., Poustka J., Hajslova J. (2010). Identification/quantification of multiple pesticide residues in food plants by ultra-high-performance liquid chromatography-time-of-flight mass spectrometry. J. Chromatogr. A.

[132] Wang J., Wei Q., Wang W., Hu H., Yan Y., Wang Y., Li Y., Jiang Y., Wu G., Hu T. (2023). Understanding the nutraceutical diversity through a comparative analysis of the taproot metabolomes of different edible radish types via UHPLC–Q–TOF–MS. Food Chem..

[133] Bauer A., Luetjohann J., Hanschen F.S., Schreiner M., Kuballa J., Jantzen E., Rohn S. (2018). Identification and characterization of pesticide metabolites in *Brassica* species by liquid chromatography travelling wave ion mobility quadrupole time-of-flight mass spectrometry (UPLC-TWIMS-QTOF-MS). Food Chem..

[134] Mu Q.E., Zhang M., Li Y., Feng F., Yu X., Nie J. (2022). Metabolomic Analysis Reveals the Effect of Insecticide Chlorpyrifos on Rice Plant Metabolism. Metabolites.

[135] Liu S., Huang Y., Liu J., Chen C., Ouyang G. (2021). In-vivo contaminant monitoring and metabolomic profiling in plants exposed to carbamates via a novel microextraction fiber. Environ. Sci. Tech..

[136] Zhao L., Huang Y., Hu J., Zhou H., Adeleye A.S., Keller A.A. (2016). ^1^H NMR and GC-MS based metabolomics reveal defense and detoxification mechanism of cucumber plant under nano-Cu stress. Environ. Sci. Tech..

[137] Ge G., Jiao W., Cui C., Liao G., Sun J., Hou R. (2019). Thiamethoxam metabolism and metabolic effects in cell suspension culture of tea (*Camellia sinensis* L.). J. Agric. Food Chem..

[138] Boonchaisri S., Stevenson T., Dias D.A. (2020). Utilization of GC–MS untargeted metabolomics to assess the delayed response of glufosinate treatment of transgenic herbicide resistant (HR) buffalo grasses (*Stenotaphrum secundatum* L.). Metabolomics.

[139] Ofoegbu P.C., Wagner D.C., Abolade O., Clubb P., Dobbs Z., Sayers I., Zenobio J.E., Adeleye A.S., Rico C.M. (2022). Impacts of perfluorooctanesulfonic acid on plant biometrics and grain metabolomics of wheat (*Triticum aestivum* L.). J. Hazard. Mater. Adv..

[140] Emwas A.H.M. (2015). The strengths and weaknesses of NMR spectroscopy and mass spectrometry with particular focus on metabolomics research. Metabonomics Methods Protoc..

[141] Scanlan L.D., Loguinov A.V., Teng Q., Antczak P., Dailey K.P., Nowinski D.T., Kornbluh J., Lin X.X., Lachenauer E., Arai A. (2015). Gene transcription, metabolite and lipid profiling in eco-indicator *Daphnia magna* indicate diverse mechanisms of toxicity by legacy and emerging flame-retardants. Environ. Sci. Technol..

